# How Well Does Climate Change and Human Health Research Match the Demands of Policymakers? A Scoping Review

**DOI:** 10.1289/ehp.1104093

**Published:** 2012-04-13

**Authors:** Jamie Hosking, Diarmid Campbell-Lendrum

**Affiliations:** 1 School of Population Health, University of Auckland, Auckland, New Zealand; 2 Department of Public Health and Environment, World Health Organization, Geneva, Switzerland

**Keywords:** climate change, environmental policy, health policy, public health, world health

## Abstract

Background: In 2008, the World Health Organization (WHO) Member States passed a World Health Assembly resolution that identified the following five priority areas for research and pilot projects on climate change and human health: health vulnerability, health protection, health impacts of mitigation and adaptation policies, decision-support and other tools, and costs of health protection from climate change.

Objectives: To assess the extent to which recently published research corresponds to these priorities, we undertook a scoping review of original research on climate change and human health. Scoping reviews address topics that are too broad for a systematic review and commonly aim to identify research gaps in existing literature. We also assessed recent publication trends for climate change and health research.

Methods: We searched for original quantitative research published from 2008 onward. We included disease burden studies that were specific to climate change and health and included intervention studies that focused on climate change and measured health outcomes. We used MEDLINE, Embase, and Web of Science databases and extracted data on research priority areas, geographic regions, health fields, and equity (systematic differences between advantaged and disadvantaged social groups).

Discussion: We identified 40 eligible studies. Compared with other health topics, the number of climate change publications has grown rapidly, with a larger proportion of reviews or editorials. Recent original research addressed four of the five priority areas identified by the WHO Member States, but we found no eligible studies of health adaptation interventions, and most of the studies focused on high-income countries.

Conclusions: Climate change and health is a rapidly growing area of research, but quantitative studies remain rare. Among recently published studies, we found gaps in adaptation research and a deficit of studies in most developing regions. Funders and researchers should monitor and respond to research gaps to help ensure that the needs of policymakers are met.

Although the risks of global climate change have been recognized for some time, interest in the specific implications of climate change for human health has grown rapidly over the past few years, with notable examples including a special issue on climate change and health published by *The Lancet* (Costello et al. 2009) and a declaration by the World Medical Association (2009). In 2008, World Health Organization (WHO) Member States passed a World Health Assembly (WHA) resolution recognizing the importance of climate change for human health, and calling for stronger commitment by Member States to address climate change–related health threats (WHA 2008). The Member States specified five research priorities that should be supported, which was an unusual and important recommendation for such a resolution to contain. The recommended priorities were

Health vulnerability to climate change and the scale and nature thereof
Health protection strategies and measures relating to climate change and their effectiveness, including cost-effectiveness
The health impacts of potential adaptation and mitigation measures in other sectors, such as marine life, water resources, land use, and transport in particular, where these could have positive benefits for health protection
Decision support and other tools, such as surveillance and monitoring, for assessing vulnerability and health impacts and targeting measures appropriately
Assessment of the likely financial costs and other resources necessary for health protection from climate change (WHA 2008).

These five priority areas were further explored in a 2009 global consultation, which resulted in further elaboration of the research needed within each priority area (WHO 2009).

Although an increasing number of papers are being published on climate change and health, the extent to which these papers match the global research priorities has not yet been assessed (WHO 2009). Moreover, although reviews of this relatively new, high-visibility and wide-ranging topic have provided useful comprehensive overviews of climate change–related health risks and opportunities (Confalonieri et al. 2007; Costello et al. 2009), we also need original research that improves our understanding of the specific pathways that link climate change to health. To assess the extent to which recently published research on climate change and health corresponds to the research priority areas identified by the Member States, we undertook a systematic review of original quantitative research on climate change and human health. Our second objective was to assess trends in the publication of climate change and health research in recent years, and the proportion of these publications that were original research rather than reviews, editorials, or other papers not based on original data.

## Methods

**Figure 1:**
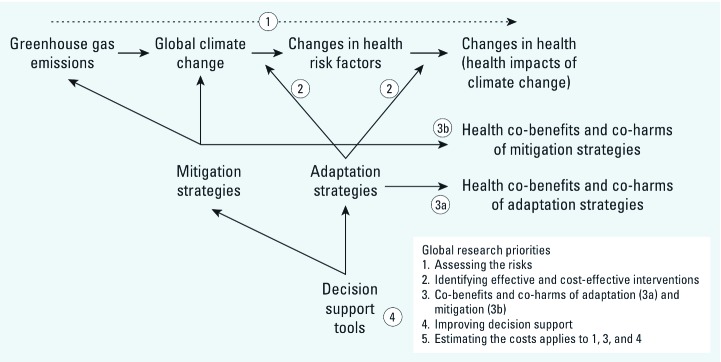
Logic model for research on climate change and health in relation to global research priorities. Categories reflect the priorities set by the WHO (2009).

*Scope of review.* Because our objective was to provide an overview of the entire field of climate change and health rather than an in-depth assessment of individual studies, we conducted a scoping review. Scoping reviews address topics that are too broad for a systematic review and commonly aim to identify research gaps in the existing literature. (Arksey and O’Malley 2005). Although we did not assess the risk of bias for individual studies (an approach commonly used by other scoping reviews), we used systematic review methods where possible to minimize bias in our identification and inclusion of studies and followed Preferred Reporting Items for Systematic Reviews and Meta-Analyses (PRISMA) standards for reporting systematic reviews and meta-analyses (Moher et al. 2009) [see Supplemental Material, Table 1 (http://dx.doi.org/10.1289/ehp.1104093)]. We developed a logic model representing links between global climate change, health, and the five research priorities, based on the WHA resolution (WHA 2008) and the report on global research priorities (WHO 2009) (Figure 1). We used this model to decide which of the five research priorities, if any, corresponded to the research topic of potentially eligible studies. We did not publish a protocol in advance of the review.

**Table 1:** Summary of characteristics of included studies.

Study characteristic/category		No. of eligible studies	Citations
Priority area					
	1) Assessing the risks		10 studies		Confalonieri et al. 2009; Doyon et al. 2008; Ebi 2008; Garg et al. 2009; Hubler et al. 2008; Husain et al. 2008; Lindgren et al. 2008; Selin et al. 2009; Tagaris et al. 2009; Tol 2008
	2) Identifying effective interventions		No studies		
	3) Co-benefits and co-harms of adaptation and mitigation		12 studies (all mitigation)		Andrae et al. 2008; Babbitt and Lindner 2008; Bloomberg et al. 2008; Friel et al. 2009; Jacobson 2008; Markandya et al. 2009; Morris and Bagby 2008; Nadal et al. 2009; Park et al. 2008; Saikawa et al. 2009; Wilkinson et al. 2009; Woodcock et al. 2009
	4) Improving decision support		10 studies		Bedsworth 2009; Bernier et al. 2009; De Schryver et al. 2009; Hahn et al. 2009; Jansen et al. 2008; Maibach et al. 2008; Nakatani et al. 2008; O’Neill et al. 2010; Sulda et al. 2010; Sundblad et al. 2009
	5) Estimating the costs		15 studies		Chapman et al. 2009; Creutzig and He 2009; Ebi 2008; Gilmore et al. 2010; Hill et al. 2009; Hubler et al. 2008; Markandya et al. 2009; Martinez 2009; Morris and Bagby 2008; Nadal et al. 2009; Pattanayak et al. 2009; Selin et al. 2009; Smith et al. 2008; Tol 2008; Tollefsen et al. 2009
Health fields					
	Multiple		19 studies		Andrae et al. 2008; Babbitt and Lindner 2008; Bedsworth 2009; Bernier et al. 2009; Chapman et al. 2009; Confalonieri et al. 2009; Creutzig and He 2009; De Schryver et al. 2009; Ebi 2008; Hahn et al. 2009; Husain et al. 2008; Maibach et al. 2008; Martinez 2009; Morris and Bagby 2008; Pattanayak et al. 2009; Smith et al. 2008; Sundblad et al. 2009; Wilkinson et al. 2009; Woodcock et al. 2009
	Air quality–related health effects		9 studies		Gilmore et al. 2010; Hill et al. 2009; Jacobson 2008; Markandya et al. 2009; Park et al. 2008; Saikawa et al. 2009; Selin et al. 2009; Tagaris et al. 2009; Tollefsen et al. 2009
	Temperature-related health effects		4 studies		Doyon et al. 2008; Hubler et al. 2008; Lindgren et al. 2008; O’Neill et al. 2010
	Vector-borne diseases		3 studies		Garg et al. 2009; Jansen et al. 2008; Tol 2008
	Noncommunicable diseases		2 studies		Friel et al. 2009; Nadal et al. 2009
	Life expectancy		2 studies		Bloomberg et al. 2008; Nakatani et al. 2008
	Nutrition		1 study		Sulda et al. 2010
World Bank income categories					
	Low- or middle-income countries (LMICs)		6 studies		Confalonieri et al. 2009; Creutzig and He 2009; Garg et al. 2009; Hahn et al. 2009; Martinez 2009; Pattanayak et al. 2009
	High-income countries (HICs)		22 studies		Andrae et al. 2008; Babbitt and Lindner 2008; Bedsworth 2009; Bernier et al. 2009; Bloomberg et al. 2008; Chapman et al. 2009; Doyon et al. 2008; Gilmore et al. 2010; Hill et al. 2009; Hubler et al. 2008; Jansen et al. 2008; Lindgren et al. 2008; Maibach et al. 2008; Morris and Bagby 2008; Nadal et al. 2009; Nakatani et al. 2008; O’Neill et al. 2010; Park et al. 2008; Sulda et al. 2010; Sundblad et al. 2009; Tagaris et al. 2009; Tollefsen et al. 2009
	Both LMICs and HICs		12 studies		De Schryver et al. 2009; Ebi 2008; Friel et al. 2009; Husain et al. 2008; Jacobson 2008; Markandya et al. 2009; Saikawa et al. 2009; Selin et al. 2009; Smith et al. 2008; Tol 2008; Wilkinson et al. 2009; Woodcock et al. 2009
WHO regions					
	AMR		13 studies		Babbitt and Lindner 2008; Bedsworth 2009; Bernier et al. 2009; Confalonieri et al. 2009; Doyon et al. 2008; Gilmore et al. 2010; Hill et al. 2009; Maibach et al. 2008; Martinez 2009; Morris and Bagby 2008; O’Neill et al. 2010; Pattanayak et al. 2009; Tagaris et al. 2009
	EUR		7 studies		Hubler et al. 2008; Jansen et al. 2008; Lindgren et al. 2008; Nadal et al. 2009; Park et al. 2008; Sundblad et al. 2009; Tollefsen et al. 2009
	WPR		5 studies		Andrae et al. 2008; Chapman et al. 2009; Creutzig and He 2009; Nakatani et al. 2008; Sulda et al. 2010
	AFR		1 study		Hahn et al. 2009
	EMR		1 study		Husain et al. 2008
	SEAR		1 study		Garg et al. 2009
	Global studies		6 studies		De Schryver et al. 2009; Ebi 2008; Jacobson 2008; Saikawa et al. 2009; Selin et al. 2009; Tol 2008
	Studies covering multiple WHO regions		6 studies		Bloomberg et al. 2008; Friel et al. 2009; Markandya et al. 2009; Smith et al. 2008; Wilkinson et al. 2009; Woodcock et al. 2009
AFR, African Region; AMR, Region of the Americas; EMR, Eastern Mediterranean Region; EUR, European Region; SEAR, South-East Asian Region; WPR, Western Pacific Region.					

*Eligibility criteria.* There was no restriction by language. Databases were searched in May and in June 2010. We included original quantitative research published in peer-reviewed journals during or after 2008. This range provided a feasible sample of current research contemporaneous with the WHA resolution. Qualitative research on this topic is also valuable, but adding this topic to our search strategy would have made it difficult to clearly define the scope of our review. Another reason we did not include qualitative research was because we considered the most active areas of policy debate in this field relate to the scale and distribution of the impacts of climate change and to the impacts of adaptation or mitigation efforts, thereby placing a premium on quantitative information.

We categorized studies under priority 1 (assessing the risks) if they studied the potential effects either of global climate change or of greenhouse gas emissions on human health (Figure 1). We included studies of global climate change (in which outcomes are compared under different global climate scenarios), but not studies of climatic events alone, because climatic events occur whether or not climate change is present. For example, we would have included a study that assessed mortality from flooding under different global climate scenarios but not a study that only assessed the effect of flooding on health.

Studies of interventions to protect health from climate change were categorized under priority 2. This category included studies of the health effects of climate change adaptation measures. We considered such studies to be eligible if the primary goal of the intervention was to protect health from climate change.

Studies of the health effects of climate change mitigation or adaptation measures were categorized under priority 3 if they focused on health effects that were not mediated by climate change. We included studies of climate change mitigation measures if they quantified changes in greenhouse gas emissions due to the intervention. For example, a study of an intervention to promote walking would be categorized under priority 3 if it assessed both effects on greenhouse gas emissions and effects on health, but not if it only assessed the effects of the intervention on health.

Under priority 4, we categorized studies that used new tools or methods that were intended to inform decisions on potential climate change interventions (including adaptation and mitigation interventions). We included studies of these tools and methods if their objective was to address the health risks of climate change. Because the report on global research priorities also included surveys of knowledge, attitudes, perceptions, and behaviors relating to health and climate change in this category (WHO 2009), we included such studies in this category.

Studies of costs relating to priorities 1, 3, or 4 were categorized under priority 5. As the WHO (2009) report addressed studies of the cost-effectiveness of interventions separately under priority 2, we assigned such studies to priority 2 for the sake of consistency.

We included studies that quantified human health outcomes using recognized health metrics, such as mortality, disease incidence or composite measures such as disability-adjusted life years. We did not include studies that only reported risk factors without quantifying health outcomes. For example, we would have included a study of the effect of global climate change on the incidence of water-borne disease, but not a study of the effect of global climate change on the frequency of heavy precipitation events alone. Health-specific outcome measures are needed to inform health policy-maker decisions in relation to other health priorities, such as resource allocation decisions. This eligibility criterion also ensured that the review remained focused on health, rather than the nonhealth impacts of global climate change.

Similarly, we required greenhouse gas outcomes to be quantified using recognized units, such as metric tonnes of carbon dioxide equivalents. Given the focus of priority 5 on costs, we also considered studies to have measured health or greenhouse gas emissions if outcomes were reported in monetary terms.

We based our definition of equity on that of Braveman and Gruskin (2003), who defined the term as

Equity in health is the absence of systematic disparities in health (or in the major social determinants of health) between groups with different levels of underlying social advantage/disadvantage— that is, wealth, power, or prestige.

We considered a study to have measured equity outcomes if it both measured differences between population groups and also assessed whether those differences were inequitable (i.e., represented systematic differences between advantaged and disadvantaged social groups). For example, we would have considered a study describing vulnerability to climate change in different countries to have addressed equity if it also analyzed which international differences, if any, were considered inequitable.

*Search strategy.* We searched MEDLINE (National Library of Medicine, Bethesda, MD, USA), Embase (Elsevier B.V., Amsterdam, the Netherlands), and Web of Science (Thomson Reuters, New York, NY, USA) databases in May and June 2010, using database limits to restrict results to original research articles published during or after 2008. Our search strategy, in summary, was (climate change or synonyms OR greenhouse gas emissions or synonyms) AND health [for details, see Supplemental Material, “Search Strategies” (http://dx.doi.org/10.1289/ehp.1104093)].

We also reviewed the reference list of a prominent review article on climate change and health (Costello et al. 2009). Although we were aware of other prominent reviews, in particular the review published by Confalonieri et al. (2007) for the Intergovernmental Panel on Climate Change (IPCC), they did not include references published during the eligible range of dates.

One researcher assessed the eligibility of all citations. Where citations could not be excluded based on title or abstract, the full-text paper was retrieved and assessed. A second reviewer independently screened a random 10% sample of citations. Any differences were resolved by discussion and led to agreement on the interpretation of study eligibility criteria.

*Data collection.* Data were extracted by one reviewer using a standardized data collection form. A second reviewer independently extracted data for a random 10% sample of included studies. Any differences were resolved by discussion and led to agreement on the interpretation of data categories.

*Data items.* We extracted data on the research priority area addressed by each study (Figure 1), the type of health outcomes, countries, and whether equity outcomes were measured by the study. Although health equity was not part of the original WHA resolution (2008), it emerged as an important theme in the subsequent WHO report describing global research priorities in this area (2009). Countries were categorized as low/middle income or high income (World Bank 2010) and by WHO region (WHO 2010).

We categorized and grouped health outcomes into health fields. Where possible, we categorized studies according to the pathway by which the health outcomes occurred (e.g., health outcomes mediated by changes in air quality, temperature, or vector-borne diseases). We identified such pathways based on previous publications that described pathways between climate change or mitigation actions and health outcomes (e.g., McMichael et al. 2006; WHO 2008). However, some studies did not focus on specific pathways. If these studies measured a specific type of health outcome, we categorized and grouped the studies according to that outcome type. If they measured multiple unrelated health outcomes, we assigned the label “multiple”.

*Synthesis of results and risk of bias.* As the objective of our review was restricted to the identification of study characteristics across a range of climate change and health topics, we made no attempt to synthesize the results of individual studies or to assess risk of bias in those studies.

*Additional analyses.* We undertook an additional analysis to assess whether, in the field of climate change and health, the proportion of publications that were original research (rather than papers not based on original data) differed from other fields. For climate change and three other topic areas (air pollution, tobacco smoking, and obesity), we analyzed the proportion of MEDLINE citations recorded as being a “journal article” (excluding review articles and commentaries), “review”, “commentary”, “editorial”, “letter”, or “news”. For these three additional topic areas, we used MEDLINE search terms based on medical subject headings such as “air pollution”, “smoking”, and “obesity”. These searches differed from the main search strategy (on climate change) because we did not develop as complex a search strategy (e.g., did not include additional “text word” searches), and we did not attempt to restrict results to studies of human health. Thus, our search strategy was not designed to detect minor differences in numbers of citations between the four topic areas. However, we considered that the searches were sufficiently similar to enable broad comparisons of numbers of citations and of citation trends. [For details, see Supplemental Material, “Search Strategies” (http://dx.doi.org/10.1289/ehp.1104093).]

The topic areas of air pollution and tobacco smoking were selected because we considered these to be well-established areas of epidemiological study. Obesity was selected because, like climate change, we considered this to be a field in which there has been growing interest in recent years. Aware that the number of papers published on climate change and health has increased rapidly in recent years (WHO 2009), we wished to test the extent to which this was specific to climate change, or reflected a more general increase in health research output. Accordingly, for each topic, we analyzed the increase in citation numbers over the past 10 years compared with a year 2000 baseline.

## Results

**Figure 2:**
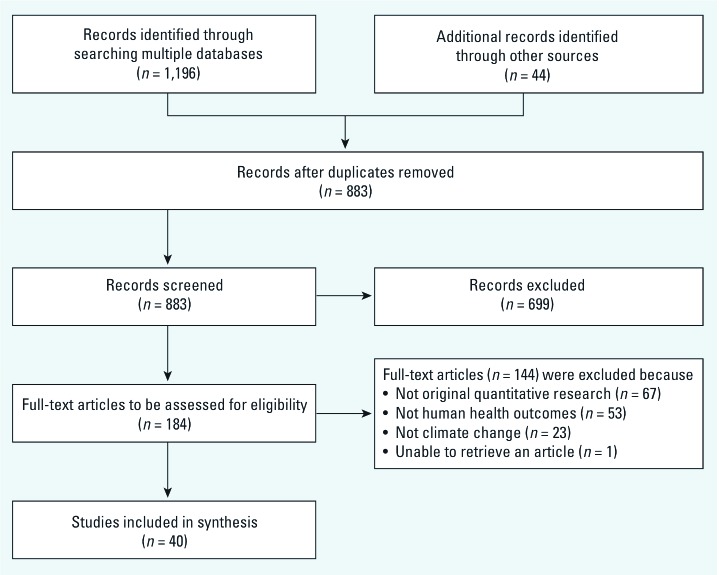
Flow diagram showing inclusion and exclusion strategy.

*Study selection.* After duplicates were removed, a total of 883 citations were identified from searches of electronic databases and review article references (Figure 2). Based on the title and the abstract, 699 were excluded, with 184 full text articles to be retrieved and assessed for eligibility. Of these, 144 were excluded for the following reasons: 23 did not directly quantify the effects of climate change, 53 did not directly quantify effects on human health, and 67 were not considered to be original quantitative research (e.g., review articles, commentaries). We excluded 1 study because we were was unable to retrieve it (Jwo et al. 2008). The remaining 40 studies were considered eligible for this review.

*Study characteristics.* Study characteristics are summarized in Table 1 and in Supplemental Material, Table 2 (http://dx.doi.org/10.1289/ehp.1104093). All studies were able to be classified under at least one of the research priority areas. Of the studies assessed, seven (Ebi 2008; Hubler et al. 2008; Markandya et al. 2009; Morris and Bagby 2008; Nadal et al. 2009; Selin et al. 2009; Tol 2008) addressed two research priorities each; all other studies addressed only one research priority each.

Global research priority areas 1, 3, 4, and 5 were each addressed by between 10 and 15 studies. However, no eligible study addressed identifying effective interventions (priority 2).

Of the studies that addressed assessing the risks (priority 1), the health fields considered included malaria (Garg et al. 2009; Tol 2008), temperature-related health risks (Doyon et al. 2008; Hubler et al. 2008; Lindgren et al. 2008), and air quality (Selin et al. 2009; Tagaris et al. 2009). Three other studies addressed multiple aspects of health (Confalonieri et al. 2009; Ebi 2008; Husain et al. 2008).

Of the 12 studies that assessed the co-benefits and co-harms of adaptation and mitigation (priority 3), all addressed the potential co-benefits of mitigation, and none dealt with adaptation strategies. Four examined co-benefits for air quality (Jacobson 2008; Markandya et al. 2009; Park et al. 2008; Saikawa et al. 2009), two examined co-benefits for noncommunicable diseases (Friel et al. 2009; Nadal et al. 2009), and the remaining studies each considered multiple aspects of health, including life expectancy (Andrae et al. 2008; Babbitt and Lindner 2008; Bloomberg et al. 2008; Morris and Bagby 2008; Wilkinson et al. 2009; Woodcock et al. 2009). Three studies in this category were life-cycle assessments that covered topics as diverse as electronics adhesives and garden-care practices (Andrae et al. 2008; Babbitt and Lindner 2008; Morris and Bagby 2008). Although not specific to climate change and health, these studies met the eligibility criteria because they quantified effects on greenhouse gas emissions or climate change, and also on health. Four studies came from a single series of papers published in *The Lancet* (Friel et al. 2009; Markandya et al. 2009; Wilkinson et al. 2009; Woodcock et al. 2009). This may have inflated the number of papers in this category during the period covered by our review, compared with other recent years.

We also assigned to priority 3 two studies that were not mitigation strategies in themselves but that were considered directly relevant to the health co-benefits of mitigation strategies. One presented an analysis showing that countries with economies that more intensively emitted greenhouse gases [expressed as carbon dioxide (CO_2_) equivalents per unit gross domestic product] had higher life expectancies (Bloomberg et al. 2008), whereas the other analyzed the effects of CO_2_ on health via effects on other air pollutants rather than effects of CO_2_ via climate change (Jacobson 2008).

We identified 10 studies on improving decision support (priority 4). Of these, 6 were surveys of knowledge, attitudes, behaviors, or preferences related to climate change and health (Bedsworth 2009; Maibach et al. 2008; Nakatani et al. 2008; O’Neill et al. 2010; Sulda et al. 2010; Sundblad et al. 2009); 2 reported results of a vulnerability assessment tool (Bernier et al. 2009; Hahn et al. 2009); 1 reported a method for estimating climate change and health impacts in life-cycle assessment (De Schryver et al. 2009); and 1 evaluated a surveillance system (Jansen et al. 2008).

A total of 15 studies assessed the monetary costs related to climate change and health (priority 5). Of these, 5 assessed costs related to vulnerability to climate change (Ebi 2008; Hubler et al. 2008; Pattanayak et al. 2009; Selin et al. 2009; Tol 2008), and 10 evaluated the costs of potential climate change mitigation strategies and their health co-benefits (Chapman et al. 2009; Creutzig and He 2009; Gilmore et al. 2010; Hill et al. 2009; Markandya et al. 2009; Martinez 2009; Morris and Bagby 2008; Nadal et al. 2009; Smith et al. 2008; Tollefsen et al. 2009), although in 1 study, climate change and health were not the primary areas of focus (Morris and Bagby 2008).

*Health fields.* Many studies (*n* = 19) measured outcomes across multiple health domains. Of those that addressed a specific health field, air quality was the most common topic (*n* = 9) (Table 1).

*Countries.* Most studies assessed health effects in high-income countries (*n* = 22) rather than low- and middle-income countries (*n* = 6) (Table 1); 12 studies assessed the effects in countries from both categories, with half of them evaluating global health effects. Each of the six WHO regions was addressed by at least 1 study. However, among the studies set in a single country, the African Region, the Eastern Mediterranean Region, and the South-East Asian Region were the setting for only 1 study each, whereas the Region of the Americas was the setting for the most studies (*n* = 13; Table 1). The most common country setting was the United States (the sole country setting for 8 studies) (Babbitt and Lindner 2008; Bedsworth 2009; Gilmore et al. 2010; Hill et al. 2009; Maibach et al. 2008; Morris and Bagby 2008; O’Neill et al. 2010; Tagaris et al. 2009).

*Equity.* No eligible studies quantitatively assessed health equity outcomes. Although several studies reported geographical differences in health effects, none of these directly assessed whether those differences were inequitable (i.e., represented systematic differences between advantaged and disadvantaged social groups).

**Figure 3:**
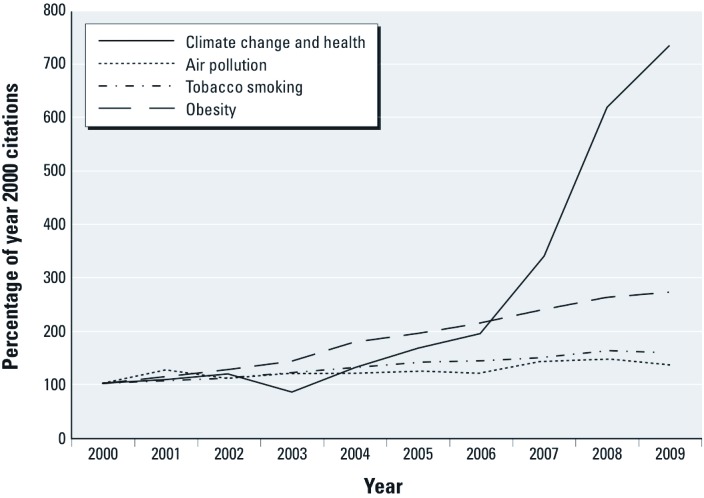
Number of MEDLINE citations, by topic, compared with year 2000 baseline.

**Figure 4:**
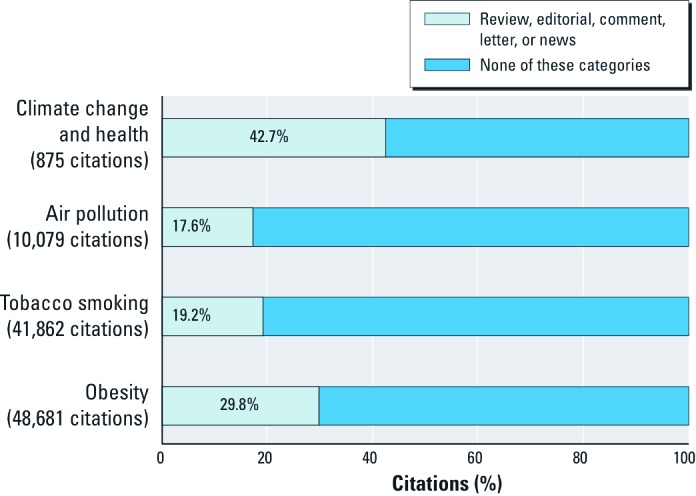
Percentage of MEDLINE citations that were not original research, 2000 to mid-2010, by topic.

*Additional analyses.* Compared with the established fields of tobacco and air pollution, the number of citations for climate change and human health increased much more rapidly over the past 10 years (Figure 3). The proportion of citations that were identified in MEDLINE as being not original research (reviews, editorials, comments, letter, or news articles) was substantially higher for climate change (43%) than for obesity (30%), air pollution (18%), and tobacco (19%) (Figure 4). This difference was largely due to the proportion of review articles, which was 29% for climate change and 20% for obesity, compared with 10–11% for the other topics. The proportion of editorials was also much higher for climate change (8%) than for the other three topics (2–3%). Note that only a small proportion of all news articles are indexed in MEDLINE.

## Discussion

Research on climate change and health is growing rapidly. A large proportion of publications on this topic consisted of reviews and editorials, which may be in part because climate change and health is a relatively new and emerging research field, although other differences between fields may also influence the proportion of reviews and editorials. Although such papers can help raise awareness of links between climate change and health, and identify promising strategies for future evaluation, more systematic and quantitative approaches are now needed.

Although the field of climate change and health is wide, the WHA resolution provides a useful structure for organizing this field that relates directly to the wishes of countries. We identified 40 studies of original quantitative research directly related to climate change and human health published in 2008 or later, covering most but not all of the research priorities identified by the WHO Member States. Although we did not include studies published before 2008, the date range of our review was appropriate for assessing the extent to which current research corresponds to global research priorities.

Our review focused on studies reporting health-specific outcomes, including mortality and morbidity and other measures of disease burden. Information on these outcomes is required to inform health policy, by indicating the importance of global climate change in relation to other health priorities. Future reviews could usefully explore specific pathways between global climate change and health in more depth, including more attention to the individual steps in each pathway.

A limitation of our review was that, due to its objective, it was restricted to an analysis of the characteristics of included studies, and did not attempt to assess the risk of bias of included studies or synthesize their results. However, we followed systematic review methods where possible to minimize bias in our identification and inclusion of studies and to ensure transparent review methods. Other systematic reviews on this topic have provided little information on the methods used or the numbers of studies identified (Swynghedauw 2009; Zhang et al. 2007).

We identified no studies assessing the effectiveness or cost-effectiveness of interventions to protect health from climate change (priority 2), and no studies of the health co-benefits or co-harms of adaptation measures (priority 3a). Interventions to adapt to, and protect health from, global climate change could have great implications for health. For example, relocating coastal communities could help protect them from grave threats such as storms and inundation, but also would be likely to have profound impacts on many of the social determinants of health, such as housing, employment, and educational opportunities. This is thus a topic of considerable importance.

The lack of studies identified by our review in this area may have been because such studies either did not directly quantify health outcomes, or because they did not self-identify as protecting health specifically from climate change. For example, studies of interventions to protect health in heat waves are present in the literature, but only met our eligibility criteria if protecting health from climate change was a specific objective of the study. This represents a weakness not only in our review but also in the link between research and policy, as decision makers seeking evidence on health adaptation strategies to climate change are likely to miss relevant information. It is also possible that researchers tend to frame studies so that they state the primary objective as addressing current needs (such as protection from heat waves) rather than more distant concerns (such as climate change adaptation), because research addressing more immediate threats may be more likely to receive funding. Nevertheless, studies of factors that mediate associations between global climate change and health, such as heat waves, need to be complemented by more analyses that quantify the health impacts mediated by these factors under different global climate scenarios.

The health effects related to air quality constituted the topic most commonly addressed by the studies in our analysis. In contrast, there were few or no studies addressing important topics such as water supplies and waterborne illness, food and malnutrition, and the health risks of extreme weather events other than heat effects. This should not be interpreted to mean that there is an absence of research in these areas, but rather that they are not well represented among recent studies that specifically link climate change and health.

Although we identified recent studies that assessed health effects in all WHO regions and in both low/middle-income countries (LMICs) and the high-income countries (HICs), LMICs were underrepresented, even though the burden of disease of climate change is expected to be highest in these countries (McMichael et al. 2004). Similarly, there were few studies in the African, Eastern Mediterranean, and South-East Asia WHO regions. This lack of research in the most vulnerable countries and regions suggests that these settings require greater priority within research agendas. However, because of limited resources in many of these countries, it is likely that expanding research in these settings will require greater use of international research collaborations (Bush et al. 2011), as well as increased funding from HICs and global agencies. Thus, the research funding agencies in the HICs may need to create dedicated funding pools for research in vulnerable countries and regions to ensure that the LMIC climate change and health proposals do not struggle to compete against national priorities set by the HICs.

Despite calls for climate change to be recognized as a priority area by health researchers, health professionals, and policy makers (Costello et al. 2009; World Medical Association 2009), the stated priorities, and the allocation decisions of those who fund health research do not always prioritize climate change research (Ebi et al. 2009). As well as directing more funding toward closing the gaps in climate change and health research, funding agencies could obtain better value within existing resource constraints by employing innovative funding strategies. Funding the development of protocols in priority research areas could be an economical way to increase the chance of researchers attracting funding from existing sources. Lessons may also be learned from the ability of competitions such as Kaggle to attract large amounts of researcher time at a relatively low cost. Kaggle (2010) is a Web platform that allows organizations to provide data relating to a specific problem, and allows international data analysis teams to compete to identify the best solution and to win cash prizes.

In addition, mechanisms to ensure that research funding decisions align with the needs of stakeholders are generally weak. This highlights the importance of both referring back to the stated requests of policymakers and also consulting relevant stakeholders to identify their needs when designing research studies. For example, the International Development Research Centre (2011) has a long-standing practice of conducting policy-relevant research at the community level, by linking scientists and affected stakeholders, from project design through implementation and evaluation phases, thereby enhancing relevance and sustainability. The same philosophy may be equally relevant at the national and international levels.

## Conclusions

Climate change and health is a rapidly growing area of research. Although recent reviews and editorials on this topic have been valuable in raising awareness of its importance, more systematic and quantitative approaches are now needed. It is also important that future research maintains good coverage of key topics—particularly the health impacts of adaptation measures—and of vulnerable geographical regions. Further monitoring and direction should help ensure that the expectations of the WHO Member States are met.

## Supplemental Material

(86 KB) PDF
