# Supplementary material for: How Well Does Climate Change and Human Health Research Match the Demands of Policymakers? A Scoping Review
Source: Environ Health Perspect. 2012 Apr 13;120(8):1076–82. doi: 10.1289/ehp.1104093 (PMC3440071; doi:10.1289/ehp.1104093)
Supplement: (86 KB) PDF [file ehp.1104093.s001.pdf]

Supplemental Material

How Well Does Climate Change and Human Health Research Match the Demands of Policymakers? A Scoping Review

Jamie Hosking, Diarmid Campbell-Lendrum

Contents

Search strategies 3
Search strategies for scoping review 3
MEDLINE (1950-present) 3
EMBASE (1947-present) 3
Web of Science (Science Citation Index Expanded 1900-present, Social Sciences Citation Index 1900-present, Arts & Humanities Citation Index 1975-present) 3
Search strategies for additional analyses (all MEDLINE, 1950-present) 4
Climate change 4
Air pollution 5
Tobacco smoking 5
Obesity 5
Supplemental Material, Table 1: PRISMA checklist 7
Supplemental Material, Table 2: Characteristics of included studies 10
References 14

Search strategies

Search strategies for scoping review

MEDLINE (1950-present)

1. climate change.tw
2. climate stabil*.tw
3. climate disrupt*.tw
4. global warming.tw
5. (greenhouse gas emission* or greenhouse-gas emission* or carbon emission* or carbon dioxide emission* or CO_2 emission*).tw
6. exp climate change/
7. or/1-6
8. health.tw
9. 7 and 8
10. limit 9 to yr="2008 -Current"
11. limit 10 to review
12. limit 10 to journal article
13. 12 not 11

EMBASE (1947-present)

1. climate change.tw
2. climate stabil*.tw
3. climate disrupt*.tw
4. global warming.tw
5. ((greenhouse gas* or CO_2 or carbon) adj2 emission*).tw
6. climate change/
7. greenhouse effect/
8. or/1-7
9. health.tw
10. 8 and 9
11. limit 10 to yr="2008 -Current"
12. limit 11 to article

Web of Science (Science Citation Index Expanded 1900-present, Social Sciences Citation Index 1900-present, Arts & Humanities Citation Index 1975-present)

Databases=SCI-EXPANDED, SSCI, A&HCI; timespan=2008-2010

1. ts="climate change"

2. ts="climate stabil*"
3. ts="climate disrupt*"
4. ts="global warming"
5. ts="greenhouse gas emission*"
6. ts="CO_2 emission*"
7. ts="carbon dioxide emission*"
8. ts="carbon emission*"
9. ts=health
10. #1 or #2 or #3 or #4 or #5 or #6 or #7 or #8
11. #9 and #10
12. #11 AND Document Type=(Article)

*Note: .tw: text word (search terms in title or abstract); .yr: year; *: wildcard; adj2: search for terms within 2 words of each other; climate change/: search for citations classified under Medical Subject Heading "climate change"; exp: explode (that is, include subheadings under the Medical Subject Heading); OR/a-n: a OR b OR c … OR n; ts=: topic (search terms in title, abstract or keywords).*

Search strategies for additional analyses (all MEDLINE, 1950-present)

Climate change

1. climate change.tw.
2. climate stabil*.tw.
3. climate disrupt*.tw.
4. global warming.tw.
5. (greenhouse gas emission* or greenhouse-gas emission* or carbon emission* or carbon dioxide emission* or CO_2 emission*).tw.
6. exp climate change/
7. or/1-6
8. health.tw.
9. 7 and 8
10. limit 9 to yr="2000 -Current"
11. limit 10 to journal article
12. limit 10 to "review"
13. limit 10 to editorial
14. limit 10 to comment
15. limit 10 to letter
16. limit 10 to news

17. or/12-16
18. 11 not 17

Air pollution

1. air pollution/ or air pollution, indoor/
2. limit 1 to yr="2000 -Current"
3. limit 2 to journal article
4. limit 2 to "review"
5. limit 2 to editorial
6. limit 2 to comment
7. limit 2 to letter
8. limit 2 to news
9. or/4-8
10. 3 not 9

Tobacco smoking

1. smoking/
2. limit 1 to yr="2000 -Current"
3. limit 2 to journal article
4. limit 2 to "review"
5. limit 2 to editorial
6. limit 2 to comment
7. limit 2 to letter
8. limit 2 to news
9. or/4-8
10. 3 not 9

Obesity

1. obesity/
2. limit 1 to yr="2000 -Current"
3. limit 2 to journal article
4. limit 2 to "review"
5. limit 2 to editorial
6. limit 2 to comment
7. limit 2 to letter
8. limit 2 to news
9. or/4-8

10. 3 not 9

*Note: .tw: text word (search terms in title or abstract); .yr: year; *: wildcard; climate change/: search for citations classified under Medical Subject Heading “climate change”; exp: explode (that is, include subheadings under the Medical Subject Heading); OR/a-n: a OR b OR c … OR n*

Supplemental Material, Table 1: PRISMA checklist

Section/topic	#	Checklist item	Reported in section
**TITLE**			
Title	1	Identify the report as a systematic review, meta-analysis, or both.	Title: scoping review
**ABSTRACT**			
Structured summary	2	Provide a structured summary including, as applicable: background; objectives; data sources; study eligibility criteria, participants, and interventions; study appraisal and synthesis methods; results; limitations; conclusions and implications of key findings; systematic review registration number.	Abstract
**INTRODUCTION**			
Rationale	3	Describe the rationale for the review in the context of what is already known.	Introduction
Objectives	4	Provide an explicit statement of questions being addressed with reference to participants, interventions, comparisons, outcomes, and study design (PICOS).	Introduction
**METHODS**			
Protocol and registration	5	Indicate if a review protocol exists, if and where it can be accessed (e.g., Web address), and, if available, provide registration information including registration number.	Scope of review; no protocol used
Eligibility criteria	6	Specify study characteristics (e.g., PICOS, length of follow-up) and report characteristics (e.g., years considered, language, publication status) used as criteria for eligibility, giving rationale.	Eligibility criteria
Information sources	7	Describe all information sources (e.g., databases with dates of coverage, contact with study authors to identify additional studies) in the search and date last searched.	Search strategy
Search	8	Present full electronic search strategy for at least one database, including any limits used, such that it could be repeated.	Supplemental material

Supplemental Material, Table 1: PRISMA checklist (cont.)

Study selection	9	State the process for selecting studies (i.e., screening, eligibility, included in systematic review, and, if applicable, included in the meta-analysis).	Search strategy
Data collection process	10	Describe method of data extraction from reports (e.g., piloted forms, independently, in duplicate) and any processes for obtaining and confirming data from investigators.	Data collection
Data items	11	List and define all variables for which data were sought (e.g., PICOS, funding sources) and any assumptions and simplifications made.	Data items
Risk of bias in individual studies	12	Describe methods used for assessing risk of bias of individual studies (including specification of whether this was done at the study or outcome level), and how this information is to be used in any data synthesis.	Not assessed
Summary measures	13	State the principal summary measures (e.g., risk ratio, difference in means).	Not assessed
Synthesis of results	14	Describe the methods of handling data and combining results of studies, if done, including measures of consistency (e.g., I^2) for each meta-analysis.	Not assessed
Risk of bias across studies	15	Specify any assessment of risk of bias that may affect the cumulative evidence (e.g., publication bias, selective reporting within studies).	Not assessed
Additional analyses	16	Describe methods of additional analyses (e.g., sensitivity or subgroup analyses, meta-regression), if done, indicating which were pre-specified.	Methods (additional analyses)
**RESULTS**			
Study selection	17	Give numbers of studies screened, assessed for eligibility, and included in the review, with reasons for exclusions at each stage, ideally with a flow diagram.	Study selection; Fig.2
Study characteristics	18	For each study, present characteristics for which data were extracted (e.g., study size, PICOS, follow-up period) and provide the citations.	Study characteristics
Risk of bias within studies	19	Present data on risk of bias of each study and, if available, any outcome level assessment (see item 12).	Not assessed
Results of individual studies	20	For all outcomes considered (benefits or harms), present, for each study: (a) simple summary data for each intervention group (b) effect estimates and confidence intervals, ideally with a forest plot.	Not assessed
Synthesis of results	21	Present results of each meta-analysis done, including confidence intervals and measures of consistency.	Not assessed

Supplemental Material, Table 1: PRISMA checklist (cont.)

Risk of bias across studies	22	Present results of any assessment of risk of bias across studies (see Item 15).	Not assessed
Additional analysis	23	Give results of additional analyses, if done (e.g., sensitivity or subgroup analyses, meta-regression [see Item 16]).	Results (additional analyses)
**DISCUSSION**			
Summary of evidence	24	Summarize the main findings including the strength of evidence for each main outcome; consider their relevance to key groups (e.g., healthcare providers, users, and policy makers).	Discussion
Limitations	25	Discuss limitations at study and outcome level (e.g., risk of bias), and at review-level (e.g., incomplete retrieval of identified research, reporting bias).	Discussion
Conclusions	26	Provide a general interpretation of the results in the context of other evidence, and implications for future research.	Discussion
**FUNDING**			
Funding	27	Describe sources of funding for the systematic review and other support (e.g., supply of data); role of funders for the systematic review.	Front page

From: Moher D, Liberati A, Tetzlaff J, Altman DG, The PRISMA Group (2009). Preferred Reporting Items for Systematic Reviews and Meta-Analyses: The PRISMA Statement. PLoS Med 6(6): e1000097. doi:10.1371/journal.pmed1000097

Protocol and registration: Note that systematic review registration and protocols are recorded by several organizations, such as the Cochrane Collaboration (www.thecochranelibrary.com)

PICOS: participants, interventions, comparisons, outcomes, and study design

For more information, visit: **www.prisma-statement.org**.

Supplemental Material, Table 2: Characteristics of included studies

Citation	Research priority area(s)	Country / countries in which health assessed	Health outcome(s)	Brief description
Andrae et al. 2008	3b	Japan	Multiple	LCA of electronics adhesives, including GHG and health effects
Babbitt and Lindner 2008	3b	United States	Multiple	LCA of re-use of coal combustion products, including GHG and health effects
Bedsworth 2009	4	United States	Multiple	Survey of public health officers about climate and health risks and preparedness
Bernier et al. 2009	4	Canada	Multiple	Results of a vulnerability assessment tool with health component
Bloomberg et al. 2008	3b	OECD countries	Life expectancy	Ecological analysis of relationship between greenhouse gas intensity of economy, and life expectancy
Chapman et al. 2009	5	New Zealand	Multiple	Cost-benefit analysis of housing insulation
Confalonieri et al. 2009	1	Brazil	Multiple	Results of a vulnerability assessment tool with health component
Creutzig and He 2009	5	China	Multiple	Models effects of congestion pricing on GHG emissions and health in monetary terms
De Schryver et al. 2009	4	Global	Multiple	Provides a method for calculating health impacts of climate change in LCA studies
Doyon et al. 2008	1	Canada	Heat and/or cold-related health effects	Models heat- and cold-related mortality using climate scenarios

Supplemental Material, Table 2: Characteristics of included studies (cont.)

Citation	Research priority area(s)	Country / countries in which health assessed	Health outcome(s)	Brief description
Ebi 2008	5, 1	Global	Multiple	Models costs of treating diarrheal disease, malaria and malnutrition for climate scenarios
Friel et al. 2009	3b	Brazil, United Kingdom	Ischaemic heart disease, stroke	Models health co-benefits of reducing emissions by less consumption of livestock products
Garg et al. 2009	1	India	Malaria	Modeling of malaria incidence using climate change scenarios
Gilmore et al. 2010	5	United States	Air quality-related health effects	Assesses health and climate change effects, in monetary terms, of batteries in electricity grid
Hahn et al. 2009	4	Mozambique	Multiple	Results of a vulnerability assessment tool with health component
Hill et al. 2009	5	United States	Air quality-related health effects	Estimates climate and health costs of biofuels and gasoline
Hubler et al. 2008	5, 1	Germany	Heat and/or cold-related health effects	Models heat- and cold-related mortality using climate scenarios
Husain et al. 2008	1	Multiple within EMR	Multiple	Models climate-related health burden in five countries near Persian Gulf
Jacobson 2008	3b	Global	Air quality-related health effects	Analyses effect of CO_2 on other air pollutants, and resulting health effects
Jansen et al. 2008	4	Germany	Vector-borne diseases	Evaluates VBD surveillance system for shifts in disease patterns from climate change

Supplemental Material, Table 2: Characteristics of included studies (cont.)

Citation	Research priority area(s)	Country / countries in which health assessed	Health outcome(s)	Brief description
Lindgren et al. 2008	1	Sweden	Heat and/or cold-related health effects	Estimates effects of climate change on health in Sweden
Maibach et al. 2008	4	United States	Multiple	Survey of public health directors on preparedness for climate change
Markandya et al. 2009	3b, 5	European Union region, India, China	Air quality-related health effects	Models air quality-related co-benefits of low-carbon electricity generation
Martinez 2009	5	Peru	Multiple	Models impacts on climate change and health, in monetary terms, from new transport system
Morris and Bagby 2008	5, 3b	United States	Multiple	LCA of natural lawn and garden care practices, including GHG and health effects
Nadal et al. 2009	5, 3b	Spain	Cancer	Cost-benefit analysis of new use of sewage sludge, including health and emissions effects
Nakatani et al. 2008	4	Japan	Life expectancy	Choice experiment for sorting of plastic waste and its climate change and health effects
O'Neill et al. 2010	4	United States	Heat and/or cold-related health effects	Survey of local government action on heat, health and climate change
Park et al. 2008	3b	United Kingdom	Air quality-related health effects	Estimates effects on health and CO_2 emissions of a fuel additive
Pattanayak et al. 2009	5	Brazil	Multiple	Models economic costs of the health effects of climate change
Saikawa et al. 2009	3b	Global	Air quality-related health effects	Models air quality-related mortality under emissions scenarios

Supplemental Material, Table 2: Characteristics of included studies (cont.)

Citation	Research priority area(s)	Country / countries in which health assessed	Health outcome(s)	Brief description
Selin et al. 2009	1, 5	Global	Air quality-related health effects	Models health and economic effects of ozone using climate scenarios
Smith et al. 2008	5	China, United States, India	Multiple	Estimates cost-effectiveness, for climate and for health, of several mitigation strategies
Sulda et al. 2010	4	Australia	Nutrition	Survey of nutritionists about link between climate and public health nutrition
Sundblad et al. 2009	4	Sweden	Multiple	Survey of knowledge in different population groups about climate change, including health effects
Tagaris et al. 2009	1	United States	Air quality-related health effects	Modeling of air quality-related mortality using climate scenarios
Tol 2008	5, 1	Global	Malaria	Models economic losses due to increased malaria incidence from climate change
Tollefsen et al. 2009	5	European Union region	Air quality-related health effects	Calculates economic benefits of accounting for GHG emissions in air quality strategies
Wilkinson et al. 2009	3b	India, United Kingdom	Multiple	Models health co-benefits of strategies to lower emissions from household energy use
Woodcock et al. 2009	3b	India, United Kingdom	Multiple	Models health co-benefits of different low-emission transport policies

EMR: Eastern Mediterranean Region; GHG: greenhouse gas; LCA: life-cycle assessment; OECD: Organisation for Economic Co-operation and Development; VBD: vector-borne disease. Global research priority areas: 1. Assessing the risks; 2. Identifying effective interventions; 3. Co-benefits and co-harms of adaptation (3a) and mitigation (3b); 4. Improving decision support; 5. Estimating the costs.

References

Andrae ASG, Itsubo N, Yamaguchi H, Inaba A. 2008. Life cycle assessment of Japanese high-temperature conductive adhesives. Environ Sci Technol 428: 3084-3089.

Babbitt CW, Lindner AS. 2008. A life cycle comparison of disposal and beneficial use of coal combustion products in Florida. Int J Life Cycle Ass 137: 555-563.

Bedsworth L. 2009. Preparing for Climate Change: A Perspective from Local Public Health Officers in California. Environ Health Perspect 1174: 617-623.

Bernier E, Gosselin P, Badard T, Bedard Y. 2009. Easier surveillance of climate-related health vulnerabilities through a Web-based spatial OLAP application. Int J Health Geogr 8: 18.

Bloomberg MR, Aggarwala RT, Bloomberg MR, Aggarwala RT. 2008. Think locally, act globally: how curbing global warming emissions can improve local public health. Am J Prev Med 355: 414-423.

Chapman R, Howden-Chapman P, Viggers H, O'Dea D, Kennedy M. 2009. Retrofitting houses with insulation: a cost-benefit analysis of a randomised community trial. J Epidemiol Community Health 634: 271-277.

Confalonieri UEC, Marinho DP, Rodriguez RE. 2009. Public health vulnerability to climate change in Brazil. Clim Res 402-3: 175-186.

Creutzig F, He DQ. 2009. Climate change mitigation and co-benefits of feasible transport demand policies in Beijing. Transport Res D-Tr E 142: 120-131.

De Schryver AM, Brakkee KW, Goedkoop MJ, Huijbregts MAJ. 2009. Characterization Factors for Global Warming in Life Cycle Assessment Based on Damages to Humans and Ecosystems. Environ Sci Technol 436: 1689-1695.

Doyon B, Belanger D, Gosselin P, Doyon B, Belanger D, Gosselin P. 2008. The potential impact of climate change on annual and seasonal mortality for three cities in Quebec, Canada. Int J Health Geogr 7: 23.

Ebi KL. 2008. Adaptation costs for climate change-related cases of diarrhoeal disease, malnutrition, and malaria in 2030. Global Health 4: 9.

Friel S, Dangour AD, Garnett T, Lock K, Chalabi Z, Roberts I, et al. 2009. Public health benefits of strategies to reduce greenhouse-gas emissions: food and agriculture. Lancet 3749706: 2016-2025.

Garg A, Dhiman RC, Bhattacharya S, Shukla PR. 2009. Development, malaria and adaptation to climate change: a case study from India. Environ Manage 435: 779-789.

Gilmore EA, Apt J, Walawalkar R, Adams PJ, Lave LB. 2010. The air quality and human health effects of integrating utility-scale batteries into the New York State electricity grid. J Power Sources 1958: 2405-2413.

Hahn MB, Riederer AM, Foster SO. 2009. The Livelihood Vulnerability Index: A pragmatic approach to assessing risks from climate variability and change-A case study in Mozambique. Global Environ Chang 191: 74-88.

Hill J, Polasky S, Nelson E, Tilman D, Huo H, Ludwig L, et al. 2009. Climate change and health costs of air emissions from biofuels and gasoline. Proc Natl Acad Sci U S A 1066: 2077-2082.

Hubler M, Klepper G, Peterson S. 2008. Costs of climate change The effects of rising temperatures on health and productivity in Germany. Ecol Econ 681-2: 381-393.

Husain T, Chaudhary JR, Husain T, Chaudhary JR. 2008. Human health risk assessment due to global warming--a case study of the Gulf countries. Int J Environ Res Public Health 54: 204-212.

Jacobson MZ. 2008. On the causal link between carbon dioxide and air pollution mortality. Geophys Res Lett 353: 5.

Jansen A, Frank C, Koch J, Stark K. 2008. Surveillance of vector-borne diseases in Germany: trends and challenges in the view of disease emergence and climate change. Parasitol Res 103: S11-S17.

Lindgren E, Albihn A, Andersson Y, Forsberg B, Olsson G, Rocklov J, et al. 2008. [Consequences of climate changes for the health status in Sweden. Heat waves and disease transmission most alarming]. Lakartidningen 10528-29: 2018-2023.

Maibach EW, Chadwick A, McBride D, Chuk M, Ebi KL, Balbus J, et al. 2008. Climate change and local public health in the United States: preparedness, programs and perceptions of local public health department directors. PLoS ONE 37: e2838.

Markandya A, Armstrong BG, Hales S, Chiabai A, Criqui P, Mima S, et al. 2009. Public health benefits of strategies to reduce greenhouse-gas emissions: low-carbon electricity generation. Lancet 3749706: 2006-2015.

Martinez MJ. 2009. Calculation of Benefits of Advanced Integrated Rail Service Application of Intelligent Transportation Systems to the Developing City of Lima, Peru. Transp Res Record2112: 26-33.

Morris J, Bagby J. 2008. Measuring environmental value for natural lawn and garden care practices. Int J Life Cycle Ass 133: 226-234.

Nadal M, Schuhmacher M, Domingo JL. 2009. Cost-benefit analysis of using sewage sludge as alternative fuel in a cement plant: a case study. Environ Sci Pollut R 163: 322-328.

Nakatani J, Aramaki T, Hanaki K. 2008. Evaluating source separation of plastic waste using conjoint analysis. Waste Manage 2811: 2393-2402.

O'Neill MS, Jackman DK, Wyman M, Manarolla X, Gronlund CJ, Brown DG, et al. 2010. US local action on heat and health: are we prepared for climate change? Int J Public Health 552: 105-112.

Park B, Donaldson K, Duffin R, Tran L, Kelly F, Mudway I, et al. 2008. Hazard and risk assessment of a nanoparticulate cerium oxide-based diesel fuel additive - A case study. Inhal Toxicol 206: 547-566.

Pattanayak SK, Ross MT, Depro BM, Bauch SC, Timmins C, Wendland KJ, et al. 2009. Climate Change and Conservation in Brazil: CGE Evaluation of Health and Wealth Impacts. BE J Econ Anal Poli 92: 6.

Saikawa E, Naik V, Horowitz LW, Liu JF, Mauzerall DL. 2009. Present and potential future contributions of sulfate, black and organic carbon aerosols from China to global air quality, premature mortality and, radiative forcing. Atmos Environ 4317: 2814-2822.

Selin NE, Wu S, Nam KM, Reilly JM, Paltsev S, Prinn RG, et al. 2009. Global health and economic impacts of future ozone pollution. Environ Res Lett 44.

Smith KR, Haigler E, Smith KR, Haigler E. 2008. Co-benefits of climate mitigation and health protection in energy systems: scoping methods. Annu Rev Public Health 29: 11-25.

Sulda H, Coveney J, Bentley M, Sulda H, Coveney J, Bentley M. 2010. An investigation of the ways in which public health nutrition policy and practices can address climate change. Public Health Nutr 133: 304-313.

Sundblad E-L, Biel A, Gärling T. 2009. Knowledge and confidence in knowledge about climate change among experts, journalists, politicians, and laypersons. Environ Behav 41: 281-302.

Tagaris E, Liao KJ, Delucia AJ, Deck L, Amar P, Russell AG. 2009. Potential Impact of Climate Change on Air Pollution-Related Human Health Effects. Environ Sci Technol 4313: 4979-4988.

Tol RSJ. 2008. Climate, development and malaria: an application of FUND. Climatic Change 881: 21-34.

Tollefsen P, Rypdal K, Torvanger A, Rive N. 2009. Air pollution policies in Europe: efficiency gains from integrating climate effects with damage costs to health and crops. Environ Sci Policy 12: 870-881.

Wilkinson P, Smith KR, Davies M, Adair H, Armstrong BG, Barrett M, et al. 2009. Public health benefits of strategies to reduce greenhouse-gas emissions: household energy. Lancet 374(9705): 1917-1929.

Woodcock J, Edwards P, Tonne C, Armstrong BG, Ashiru O, Banister D, et al. 2009. Public health benefits of strategies to reduce greenhouse-gas emissions: urban land transport. Lancet 374(9705): 1930-1943.
